# Case report: Comprehensive clinical, pathological and genetic investigations to decipher the background of cyclic thrombocytopenia

**DOI:** 10.3389/pore.2024.1611914

**Published:** 2024-09-30

**Authors:** Zsófia Flóra Nagy, Kristóf Árvai, Péter Lakatos, Ildikó Beke Debreceni, Balázs Szili, Ildikó Istenes, Csaba Bödör, Judit Demeter

**Affiliations:** ^1^ Department of Internal Medicine and Oncology, Faculty of Medicine, Semmelweis University, Budapest, Hungary; ^2^ Department of Laboratory Medicine, Faculty of Medicine, University of Debrecen, Debrecen, Hungary; ^3^ Hungarian Centre of Excellence for Molecular Medicine (HCEMM)-Semmelweis University (SE) Molecular Oncohematology Research Group, Department of Pathology and Experimental Cancer Research, Semmelweis University, Budapest, Hungary

**Keywords:** case report, WES, cyclic thrombocytopaenia, thrombocytopaenia, cyclic

## Abstract

Cyclic thrombocytopenia (CTP) is a rare disease characterized by the oscillations seen in the platelet count of the patients. The pathomechanism of the disease is poorly understood, several pathological processes have been implied in the background of CTP. In our current study, we aimed to thoroughly investigate the case of a 41-year-old female patient with a 22-year history of CTP. Wide-ranging laboratory testing, histological analyses and genetic investigations were carried out to investigate all the defects and alterations of physiological pathways described in the background of CTP to date. Bone marrow biopsy showed normal hemopoiesis with the abundance of megakaryocytes, some of which displayed hypolobulated nuclei. T-cell receptor rearrangement studies showed a polyclonal pattern with no indication of a monoclonal cell population. Flow cytometric assessment of the platelets revealed large number of immature platelets and decreased expression of glycoprotein IIb and IIIa at platelet zenith. Increased expression of glycoprotein IIb, IIIa and glycoprotein Ib-IX complex was observed at the nadir of the cycle. Whole exome sequencing revealed a heterozygous missense variant of uncertain significance in the SERPINC1 gene, which has been associated with hereditary antithrombin deficiency. The screening of autoantibodies did not reveal signs of autoreactive processes, and no thyroid dysfunction was found. Furthermore, synchronization with the menstrual cycle could not be concluded based on our patient’s case. With our results we contribute to the very limited data known about the long-term course of the disease and provide valuable insights into the genetic architecture of CTP.

## Introduction

Cyclic thrombocytopenia (CTP) is a disease characterized by the regular oscillations of the platelet count between dangerously low and normal or dangerously high levels. During periods of severe thrombocytopenia, life threatening bleedings may occur, whilst at the zenith of the cycle the risk of thrombotic events rises [1]. The condition is very rare, probably often misdiagnosed as immune thrombocytopenia (ITP) [2]. Currently, the exact cause of CTP is unknown, intensive research is aimed to uncover CTP associated factors which may bring us closer to understanding the pathophysiology behind the disease [3]. The therapeutic approaches reported until now have reached remission only in exceptional cases, individual successes have been described [4, 5].

## Patient

Our current study was performed in accordance with the principles of the Declaration of Helsinki. The patient gave her informed consent to participation in the investigation and our study was approved by the ethical board (BMEÜ/2864- 1/2022/EKU).

Hereby we report the case of a currently 41-year-old female patient, born in 1981. In her past medical history strabismus correction surgeries at the age of 5 and 7 years. At the age of 18 years, she experienced prolonged and heavy menstrual bleeding and hypochromic microcytic anaemia was seen in her laboratory values. Thus, she was referred to our hematology department, where normal white blood cell count and differential but severe thrombocytopenia were noted.

Upon serological examination irregular antibodies were detected in the patient’s blood, which consisted of enzymatic panantibodies and cold type auto anti-I specific antibodies. Hepatitis B and C and HIV serologies were negative. Thus, her anaemia was suspected to be of immunologic origin, and the diagnosis of Evans syndrome was set up based on the autoimmune hemolytic anaemia and thrombocytopenia. Upon physical examination her spleen was not enlarged. Steroids (even in large doses) did not achieve a satisfactory response nor in relationship to the anaemia neither to the thrombocytopaenia. The patient had to be hospitalized several times because of life threatening bleedings over the course of the next year. In the following year splenectomy was performed with premedication with intravenous immunoglobulin and iv. vincristine. After the splenectomy the hemolytic anaemia gradually ceased. However, the thrombocytopenia persisted even after the surgery, thus cyclosporin therapy was initiated, which lead to the vast improvement of the platelet count. Over the next 4 years, she presented with acute thrombocytopenic shubs several times at our department and cyclosporin therapy was used and it led to quick recovery each time. From the age of 24, cyclosporin had to be augmented with iv immunoglobulins due to the gradually decreasing efficacy of cyclosporin. Two years later, at the age of 26 the patient refused to take any more cyclosporin due the adverse effects she experienced (depression and stomach pain).

**FIGURE 1:**
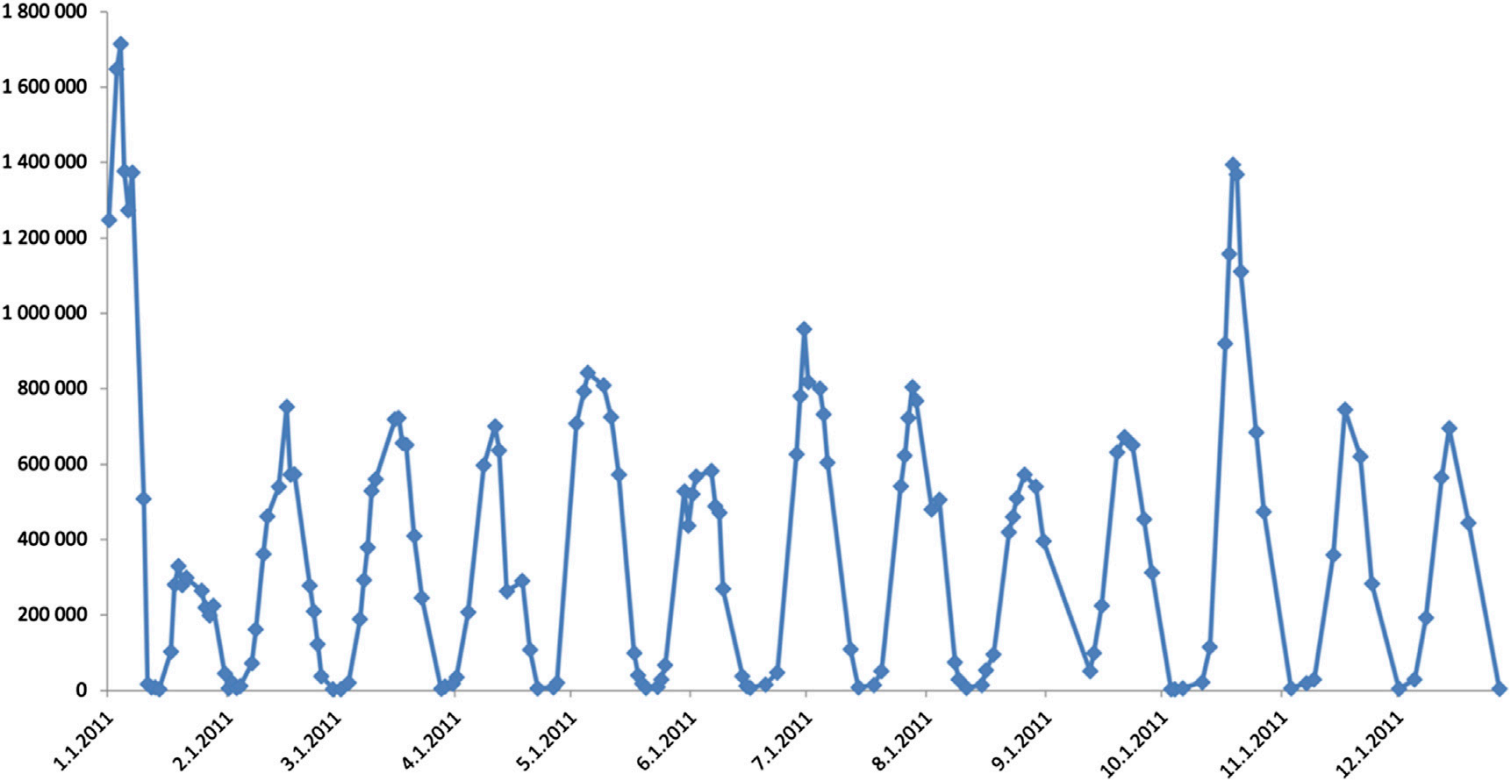
Platelet count oscillations in 2011. Platelet count is represented by *Y*-axis values, while *X*-axis shows the date.

At the age of 29, romiplostim therapy was started with a weekly dose of 1 ug/kg. 3 weeks after the initiation of romiplostim, the patient complained of shortness of breath and chest pain. At this time her platelet counts reached 1600 G/L. On ECG examination negative T waves were seen, but cardiac necroenzyme elevations were absent. A consult with cardiology suspected the complaints to originate from a hypervolemic state. From this time on, it was noticed that her platelet count fluctuated between extreme values without any external stimuli. It was observed that her platelet count followed a 21-day cycle with the minimum platelet number being below 5 G/L (at most critical times the platelet count was even counted from blood smear, not only measured with a laboratory automat) and maximum reaching way over 1,500 G/L. Figure 1 shows the oscillations of the platelet count in the calendar year of 2011.

Based on the literature, 100 mg rituximab was administered intravenously weekly, altogether three times [6]. After the third infusion she developed thrombophlebitis and rituximab was discontinued because of the complication of the infusion and the lack of efficacy until that time.

Since then, the patient has been attending regular checkups and she keeps track of the fluctuation of her platelet counts. During platelet nadirs the patient experiences epistaxis, gastrointestinal, mucocutaneous, umbilical and gynecological bleeding. In times, when her menstrual cycle coincides with the platelet nadir, norethisterone was used to suppress her menstrual bleeding. 3 months ago she has been switched to continuous dosing of norethisterone, and since then she did not experience menstrual bleedings. Whenever she entered the rising part of the cycle, she took 100 mg/die of acetylsalicylic acid to prevent clogging. Symptoms during these times include headaches. She has had low vitamin B12 levels in the past, thus her B12 levels are regularly checked and supplemented if needed. Currently her LDH is only slightly elevated (up to 350 U/L) and no other laboratory sign points to an ongoing hemolysis.

Table 1 shows the treatment history of our patient.

**TABLE 1:** Treatment history of our patient.

Treatment number	Drug/intervention	Dose	Duration	Response	Reason for discontinuation
1	Methylprednisolone	125 mg and up to 1,000 mg iv	Several days, altogether several weeks	Not satisfactory	Lack of response and steroid associated side effects (steroid myopathy)
2	Dexamethasone	iv 40 mg daily for 4 days	Occasional, only during low platelet phases	Not satisfactory	Not effective enough, steroid associated adverse effects
3	Open splenectomy	—	No surgical complications were noted	Hemolytic anaemia gradually ceased but not satisfactory response with regard to thrombocytopaenia	—
4	Vincristin	iv 1 mg/m^2^ biweekly	3 months (2000 January-March)	Initially good but decreased with time	Decreasing effecitivity
5	Cyclosporin	3 mg/kg	62 months (2000 March–2005 May)	Temporarily very good but decreased with time	Depression, stomach ache
	Cyclosporin+ IvIG	3 mg/kg (cyclosporin) + 10 g (IvIG)	24 months (2005 May-2007 May)	Initially good but decreased with time	Depression, stomach ache
6	Romiplostim	1 ug/kg weekly	3 weeks (2011 January)	Extreme thrombocytosis	Chest pain and shortness of breath
7	Rituximab	100 mg iv weekly	3 weeks	Not satisfactory	Thrombophlebitis
8	Acetylsalicylic acid	100 mg per os	Only in the thrombocytosis phases	Clinically satisfactory	—

There is an abundance of early deaths due to cardial thromboembolic events in the patient’s family; her mother passed at the age of 56, her father and paternal grandfather exited at the age of 65, all of acute myocardial infarction.

## Methods

As with the years passing more of the possible causes of CTP have gotten published in the scientific literature and testing of the described parameters became available, more and more workup was carried out.

Different laboratory parameters were measured by the Department of Laboratory Medicine, Semmelweis University from adequately anticoagulated venous blood samples. The patient has also undergone a bone marrow biopsy of the superior iliac spine according to institutional standard practices and immunohistochemical reactions staining for CD61 antigen (BioSB CD61 2f2 antibody, BioSB, Santa Barbara, United States). Analysis of the biopsy specimen and T-cell receptor rearrangement investigations were carried out in 2018 in the Department of Pathology and Experimental Cancer Research, Semmelweis University.

For the flow cytometric examination of glycoproteins (GPs) the following antibodies were used CD 41 PE (Dako, Glostrup, Denmark), CD42a FITC, CD42b FITC and CD61 PE (Becton Dickinson, Mansfield, MA, United States). During the analysis, platelets were identified based on their light scattering properties, and the expression of GPs was determined based on the mean fluorescence intensity (MFI) of fluorescent-labeled antibodies. The analyses were performed on a BD FACS-Canto II instrument (Becton Dickinson, Mansfield, MA, United States), and data were analyzed via BD FACSDiva software (6.1.3 version).

For the next-generation sequencing venous blood sample was taken from the patient and then genomic DNA was isolated. Twist exome 2.0 kit was used for exome capturing and next-generation sequencing was performed on Illumina Novaseq device. Sequences were compared to hg38 and variant calling was executed on Illumina Dragen platform. For interpretation of variants and for assessment of their significance Franklin by genoox^1^ and other predictive platforms were used. Classification of the variants was performed according to the guidelines of the American College of Medical Genetics and Genomics (ACMG) [7]. For the data analysis several virtual panels were created, and we analyzed the sequencing data by following these panels. First filtering criteria was custom designed “comprehensive hematology and immunology” panel which contained 749 genes (Supplementary Table 1). Next up we used phenotypic driven approach based on HPO terms (HP:0011873, HP:0001894, HP:0001873, HP:0011875, HP:0001872). To surely not miss a relevant variant we filtered the variants based on their submission as pathogenic or likely pathogenic to the ClinVar database. Lastly, we checked the ACMG Secondary Findings V3.2. After identifying our variant of interest, we conducted a targeted analysis of the genes of the Serpin family. All revealed VUSs or stronger variants are listed in Table 2.

**TABLE 2:** VUS-s and stronger variants detected by WES in our patient.

Gene	Transcript	AA Change	Nucleotide	dbSNP	Zygosity	REVEL Score	Condition (OMIM)
AK2	NM_001625.4	p.Lys181Asn	c.543G>C	rs72884305	het	0.192	Reticular dysgenesis (# 267500)
AK2	NM_001625.4	p.Gly205Glu	c.614G>A	rs202182972	het	0.817	Reticular dysgenesis (# 267500)
BACH2	NM_021813.4	p.Glu209Gln	c.625G>C		het	0.021	Immunodeficiency 60 and autoimmunity (# 618394)
CFTR	NM_000492.4	p.Arg31Cys	c.91C>T	rs1800073	het	0.669	Cystic fibrosis (# 219700)
CFTR	NM_000492.4		c.1210-12_1210-11delTT	rs1805177	het		Cystic fibrosis (# 219700)
FANCD2	NM_001018115.3		c.1278+1delG	rs750338758	het		Fanconi anemia, complementation group D2 (# 227646)
FTCD	NM_206965.2	p.Thr453Met	c.1358C>T	rs200283734	het	0.117	Glutamate formiminotransferase deficiency (# 229100)
HAVCR2	NM_032782.5	p.Thr101Ile	c.302C>T	rs147827860	het	0.278	T-cell lymphoma, subcutaneous panniculitis-like (# 618398)
KMT2D	NM_003482.4	p.Met4001Ile	c.12003G>A		het	0.22	Kabuki syndrome 1 (# 147920)
MEFV	NM_000243.3	p.Lys695Arg	c.2084A>G	rs104895094	het	0.353	Familial Mediterranean fever (# 249100)
NSMCE2	NM_173685.4	p.Ala118Val	c.353C>T		het	0.12	Seckel syndrome 10 (# 617253)
OFD1	NM_003611.3	p.Arg799Pro	c.2396G>C		het	0.215	Simpson-Golabi-Behmel syndrome, type 2 (# 300209)
RAD50	NM_005732.4	p.Gln404fs	c.1208_1209dupGA	rs786203655	het		Nijmegen breakage syndrome-like disorder (# 613078)
SEC23B	NM_006363.6	p.Ala536Gly	c.1607C>G	rs535355543	het	0.81	Dyserythropoietic anemia, congenital, type II (# 224100)
SERPINC1	NM_000488.4	p.Thr10Asn	c.29C>A	rs61736655	het	0.141	Thrombophilia 7 due to antithrombin III deficiency (# 613118)
SLC4A1	NM_000342.4	p.Arg155Trp	c.463C>T	rs536419419	het	0.603	Spherocytosis, type 4 (# 612653)

We used the custom designed “comprehensive hematology and immunology” panel and filtered for relevant variants. Pink coloring of the condition column means that the zygosity of the detected variants does not fit the inheritance mode of the condition. het, heterozygous.

## Results and discussion

A thorough search of literature revealed possible laboratory and clinical characteristics of CTP patients which provide valuable insights into the proposed pathomechanism of the disease. These pathological pathways might not be exclusive and several mechanisms may lay behind such a complex condition [3].

Autoreactive antibodies and autoimmune diseases show an increased incidence among CTP patients, with the thyroid gland being the most frequently affected organ [8]. Thyroid hormone levels and TSH were in the normal range in our patient, thyroid specific autoantibodies were not present. Upon physical examination the thyroid gland was not found to be enlarged, thus we omitted the ultrasound examination. The patient did not report any concomitant diseases except for a mild hypertension. Additional antibodies were screened (SMA, ANA, AMA, antidsDNA, ENA, SS-A-Ro, SS-B-La, anti RNP antibody, Scl-70 antibody, Jo-1 antibody) with negative results.

Several reports describe the platelet oscillations to be in sync with the menstrual cycle of the patients [9]. Furthermore, variation was seen regarding which part of the platelet cycle overlaps with the menstrual cycle; both platelet zeniths and nadirs were seen at the time of ovulation as well [9, 10]. No synchronization could be observed in the case of our patient, in 2021 five of her menstrual cycles coincided with the nadir of her platelet counts which necessitated the use of norethisterone to prevent life threatening consequences. This may originate from the fact that the patient’s platelet cycle is fairly regular, lasting 21 days and does not seem to alter because of external factors. Since using norethisterone continuously the patient’s menstrual bleedings have completely stopped.

**FIGURE 2:**
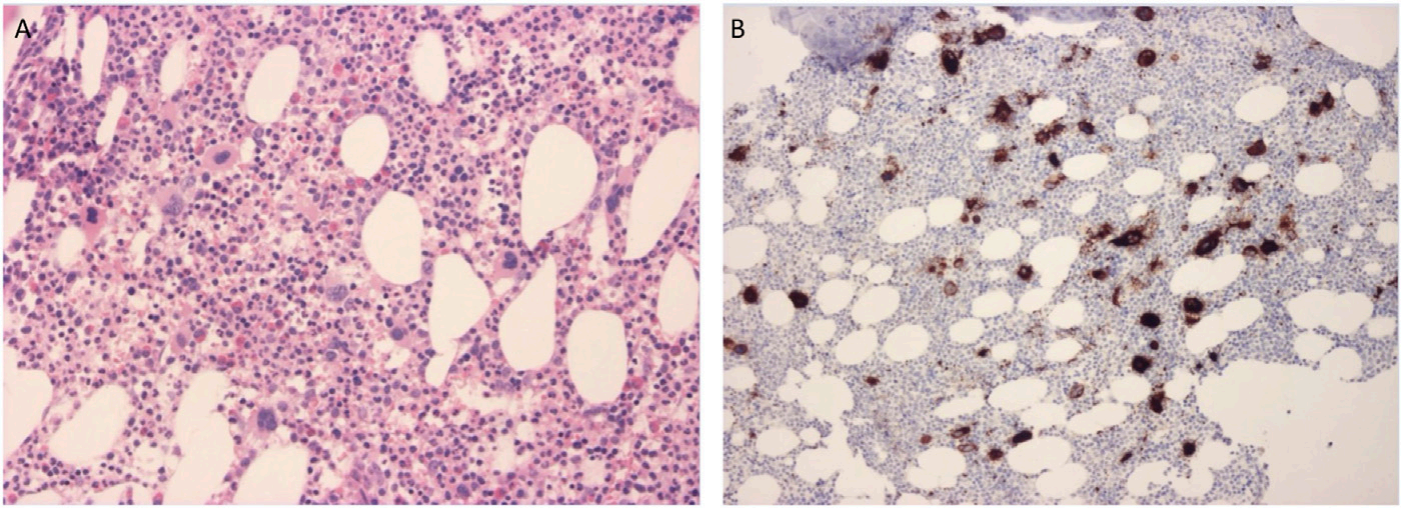
**(A)** Bone marrow biopsy specimen stained with hematoxylin-eosin shows an abundance of megakaryocytes. **(B)** Bone marrow biopsy specimen stained with CD61 immunohistochemistry marking megakaryocytes. Micromegakaryocytes did not stain with the marker.

For the possible identification of changes and disruptions in hemopoiesis a bone marrow biopsy from the superior posterior iliac spine was performed in 2011 and in 2018. Abnormal thrombocytopoesis may be a feature of CTP [11]. The biopsy in 2011 was performed at a severely thrombocytopenic time point, while at the time of the one, 7 years later the patient’s platelet count was within the normal range. Both samples gave similar results and showed normal hemopoiesis with a cellularity of 60%–70%. The structure of the bone marrow was intact. Reactive megakaryocyte outgrowth was observed which showed typical cytomorphology and did not form clusters. The number of CD61 positive megakaryocytes was the double of the normal value. Most of them were significantly smaller than the normal (micromegakaryocytes) and bared mild dysplastic features such as a hypolobulated nucleus. The micromegakaryocytes did not show staining with the CD61 marker. Fibrosis could not be detected neither morphologically nor with silver staining. Figure 2 shows the results of the 2018 bone marrow biopsy.

Antiplatelet antibodies might play a role in the increased destruction of platelets [12]. However, in our patient no antiplatelet antibodies could be detected.

**FIGURE 3:**
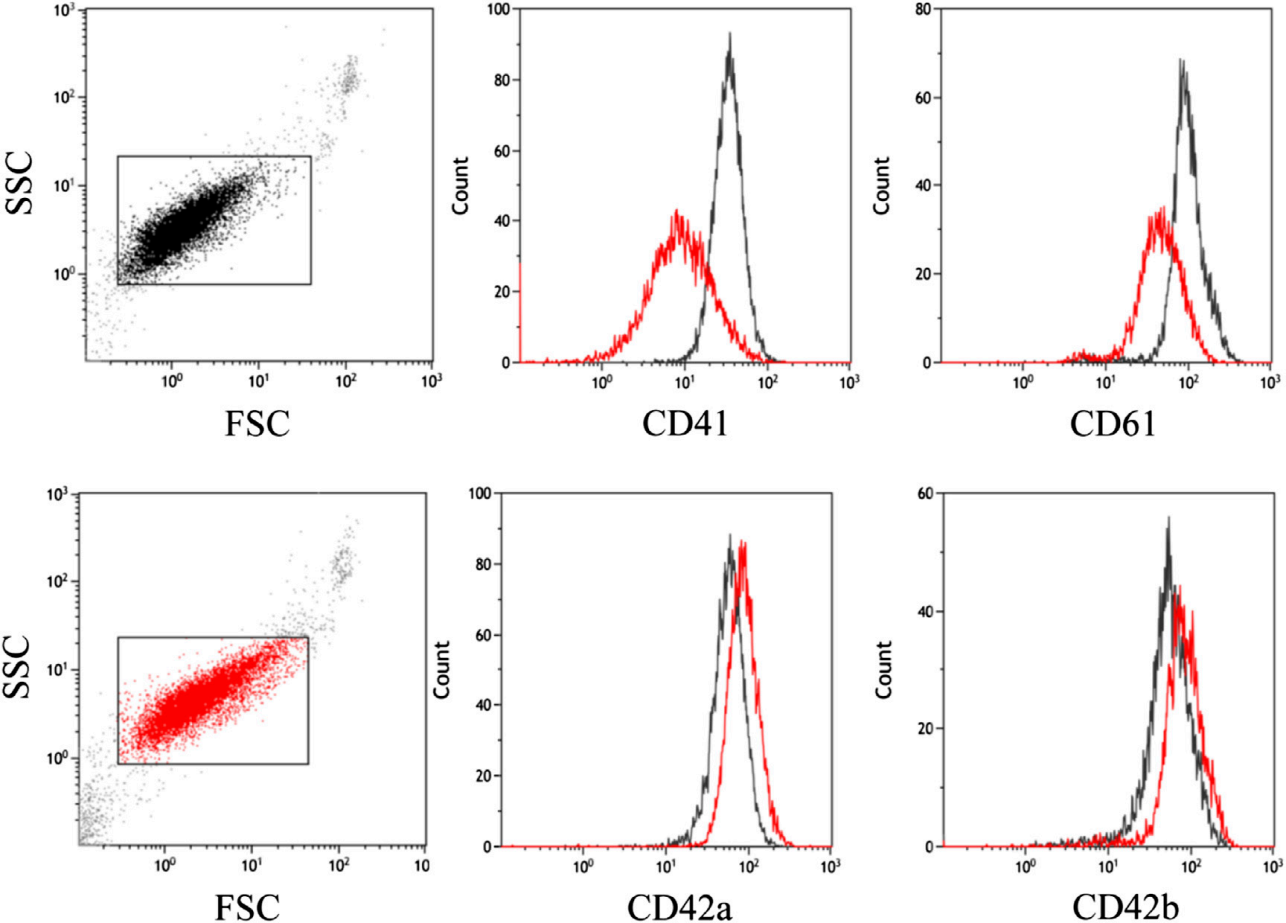
Flow cytometric examination of platelet glycoproteins. Control (in black) and patient (in red) platelets were stained with CD42a (GPIX), CD42b (GPIb), CD41 (GPIIb) and CD61 (GPIIIa) antibodies. Single platelets are in a rectangular gate on the FSC-SSC dot-plot. The patient’s platelets (in red) have a higher FSC value, which indicates a larger cell size. Histograms show lower mean fluorescence intensity (MFI) of CD41 and CD61 and higher CD42a and CD42b MFI on patient platelets.

Flow cytometric examinations were performed at platelet zenith and at platelet nadir, around 1 month apart to measure platelet surface glycoproteins (see Figure 3). Findings of the measurement performed at peak platelet count showed enlarged platelets, around double in size of a normal platelet. Immature platelet fraction accounted for 19.4% of platelets (ref: 1.4%–7.9%). CD41 fluorescence values showing surface GP-IIb were measured at around 1/3 of the normal intensity and mean fluorescence intensity of GP-IIIa was around half of the control values. CD42b and CD42a fluorescence values (representing GP-Ib and GP-IX respectively) were found to be 1.5 times higher than of the controls. The large fraction of immature platelets could be held accountable for the decreased expression of GP-IIb and GP-IIIa markers.

Repeating the same flow cytometric measurement when the number of platelets was only 6 G/L revealed significantly larger platelets, and all glycoprotein marker levels were elevated (GP-Ib and GP-IX showed a fivefold increase to the control and GP-IIb and GP-IIIa showed doubled levels). The immature platelet fraction was measured 8.4%, which is still higher than the reference value. The larger platelets and elevated glycoprotein expression is characteristic of activated platelets, which could lead to their consumption.

Ours is the first study to our knowledge to characterize the expression of glycoprotein markers in depth in the case of cyclic thrombocytopenia. It would of great importance to compare and contrast our findings with other groups’ results.

Clonal T-cell mediated pathological mechanisms may contribute to the fluctuations of the platelets [8]*.* T-cell receptor rearrangements have been identified as a causal alteration behind CTP in less than 10 patients worldwide [8, 13, 14]. Füreder et al., were the first to describe T-cell receptor rearrangements as the causative defect of CTP. The neutrophil and reticulocyte count of the patient described in their report also showed oscillations [14]. In the case of our patient, T-cell populations and T-cell receptor rearrangements were examined on the 2018 bone marrow biopsy sample and from the peripheral blood. The findings showed polyclonal T-cell receptor rearrangements in both investigated regions (VgIf/Vg10 - Jg1.1/2.1/1.3/2.3 and Vg9/Vg11 - Jg1.1/2.1/1.3/2.3). Moreover, in our patient’s neutrophil and reticulocyte levels stayed within normal range.

Hematological malignancies and chronic myeloproliferative diseases may present with thrombocytopenia. Kojima et al., identified cyclic platelet oscillations in a patient diagnosed with polycythemia vera [15]. An exceptional CTP case in a patient with polycythemia vera was provoked by hydroxyurea treatment, although the trigger remained unclear [16]. To exclude the possibility of an undiagnosed polycythemia vera in our patient, extensive investigations were carried out. Mutational screening of the *JAK2* p.V617F variant, the *calreticulin* (*CALR*) and *MPL Proto-Oncogene, Thrombopoietin Receptor* (*MPL*) genes have returned the wild type sequence. Over the more than 22 years of longitudinal observation, the patient did not show signs of a malignant transformation. However, the occurrence of malignancies in the future cannot be ruled out, thus we will continue to watchfully follow the course of the patient’s condition.

Lastly, a whole exome sequencing (WES) approach was used to identify the potential underlying genetic cause of CTP. During the analysis of WES data, no relevant pathogenic or likely pathogenic variants were uncovered. A rare missense variant was uncovered in the *SERPINC1* gene. According to the ACMG guidelines the sequence alteration is categorized as a variant of uncertain significance and fulfills the PM2 pathogenic moderate (cut off value used: 0.109%, which is the frequency of the most common pathogenic variant in this gene) and PP2 pathogenic supporting (based on the number of pathogenic missense variants in the gene) criteria [7]. The variant in the *SERPINC1* gene (NM_000488.4: c.29C>A) changes the 10th amino acid of the protein from the wild type threonine to asparagine. The variant is very rarely identified in the most relevant population genetics databases, homozygous individuals have not been reported to date. The variant has been submitted to ClinVar four times,^2^ two submissions stating a likely benign effect, while two other credible submissions consider it a variant of uncertain significance. The Thrombosis Variant Curation Expert Panel has also curated the variant classification and report it as a likely benign sequence alteration in association with thrombotic events. However, in our case the variant surfaced in connection with cyclic thrombocytopaenia and not thrombosis. Although the REVEL score of our variant is 0.141, which predicts a benign effect, the whole-genome annotator GenoCanyon predict its deleterious effect on the protein [18].

The p.Thr10Asn variant is located in the signal region of the protein which is cleaved from the mature protein product as it passes through the endoplasmic reticulum [18]. Thus, we may suppose that the variant interferes with the physiological maturation process of the protein or cannot be removed and the signal peptide may be retained in the secreted protein as well. The protein encoded by the *SERPINC1* gene belongs to the serpin family and acts as a serin protease inhibitor. Antithrombin III is an inhibitor of thrombin, thus restricts the activity of the factors IXa, Xa, XIa [19].

After the identification of the p.Thr10Asn variant in the *SERPINC1* gene a complex thrombophilia testing commenced. Blood was drawn at normal platelet levels and a thrombophilia laboratory panel was performed. Antithrombin antigen level was determined to be 0.27 g/L (reference range: 0.19–0.31 g/L). Activity assessment of antithrombin III yielded 98%, thus falls in the normal range. Interestingly, the activity of coagulation factor VIII was 398.5%, which is two times the upper limit of the normal range according to our laboratory. With our results, we do not support the recategorization and prioritization of the p.Thr10Asn variant, it is unlikely that the variant is causal to CTP. Detailed functional studies will be needed in the future to confirm our findings and to unravel the exact effect of the variant on the function of antithrombin III.

Family testing was not available to confirm the *de novo* origin of the variant, because both parents of the patient had already passed and the patient has no siblings. However, it may be assumed that the variant could have led to the cumulative incidence of the early cardiovascular deaths in the patient’s family, while the role of environmental and lifestyle factors cannot certainly be excluded.

## Conclusion

In our current work, we performed the detailed diagnostic investigations in a patient with a long history of CTP. All factors associated with CTP reported in the literature were examined. Furthermore, whole exome sequencing was performed to assess the possible genetic background of the disease.

With our results we wish to contribute to sparse data available about the pathogenesis of CTP. With the growing number of cases investigated, a better understanding of the pathophysiology behind CTP might be reached.

To the best of our knowledge, ours is the first study to utilize a whole exome sequencing approach to investigate the genetic background of CTP. With next-generation sequencing techniques being widely available we hope to see more and more CTP patients genetically characterized. Larger genetic aberrations of the genome were not screened in our study, future research directions should be aimed to examine the copy number variations associated with CTP. Additionally, the gene or genes behind CTP may not have been identified yet, and reanalysis of the currently acquired data should be performed if a gene of interest emerges in the scientific literature. In the future genome wide association studies will be needed on larger cohorts to better understand the genetic background of cyclic thrombocytopenia.

## Data Availability

Publicly available datasets were analyzed in this study. This data can be found here: file will be made available upon request.

## Footnotes

1
https://franklin.genoox.com/

2
https://www.ncbi.nlm.nih.gov/clinvar/variation/870846
