# Supplementary material for: Case report: Comprehensive clinical, pathological and genetic investigations to decipher the background of cyclic thrombocytopenia
Source: Pathol Oncol Res. 2024 Sep 30;30:1611914. doi: 10.3389/pore.2024.1611914 (PMC11471597; doi:10.3389/pore.2024.1611914)
Supplement: Supplementary file 1 [file DataSheet1.pdf]

Gene list of the custom designed "comprehensive hematology and immunology panel"

ABCA3	*CD93*	*F13B*	*IL17RA*	*NCF2*	*RHCE*	*SPTB*
ABCB7	*CDAN1*	*F2*	*IL17RC*	*NCF4*	*RHOH*	*SRC*
ABCG5	*CDC42*	*F5*	*IL18BP*	*NCKAP1L*	*RIPK1*	*SRP54*
ABCG8	*CDCA7*	*F7*	*IL1RN*	*NCSTN*	*RIT1*	*SRP72*
ACD	*CDK9*	*F8*	*IL21*	*NDUFB11*	*RMRP*	*STAT1*
ACP5	*CDKN2A*	*F9*	*IL21R*	*NEUROG3*	*RNASEH2A*	*STAT2*
ACTA2	*CEBPA*	*FAAP100*	*IL23R*	*NFAT5*	*RNASEH2B*	*STAT3*
ACTB	*CEBPE*	*FAAP24*	*IL2RA*	*NFE2L2*	*RNASEH2C*	*STEAP3*
ACTN1	*CENPF*	*FADD*	*IL2RB*	*NFIL3*	*RNF168*	*STIM1*
ACVRL1	*CFAP298*	*FANCA*	*IL2RG*	*NFKB1*	*RNF31*	*STK36*
ADA	*CFAP300*	*FANCB*	*IL36RN*	*NFKB2*	*RNU4ATAC*	*STK4*
ADA2	*CFB*	*FANCC*	*IL6R*	*NFKBIA*	*RORC*	*STN1*
ADAM17	*CFD*	*FANCD2*	*IL7R*	*NHEJ1*	*RPA1*	*STX11*
ADAMTS13	*CFHR5*	*FANCE*	*INO80*	*NHP2*	*RPGR*	*STX3*
ADAMTS3	*CFI*	*FANCF*	*IRAK1*	*NLRC4*	*RPL10*	*STXBP2*
ADAR	*CFP*	*FANCG*	*IRAK4*	*NLRP1*	*RPL11*	*STXBP3*
ADH5	*CFTR*	*FANCI*	*IRF2BP2*	*NLRP12*	*RPL15*	*TAP1*
ADIPOQ	*CHD7*	*FANCL*	*IRF3*	*NLRP3*	*RPL18*	*TAP2*
ADIPOR2	*CHST14*	*FANCM*	*IRF4*	*NME8*	*RPL19*	*TAPBP*
AICDA	*CIB1*	*FAS*	*IRF7*	*NOD2*	*RPL26*	*TASP1*
AIRE	*CIITA*	*FASLG*	*IRF8*	*NOP10*	*RPL27*	*TAZ*
AK1	*CLCN7*	*FAT4*	*IRF9*	*NOTCH1*	*RPL31*	*TBK1*
AK2	*CLEC7A*	*FBN1*	*ISG15*	*NPM1*	*RPL35A*	*TBX1*
ALAS2	*CLPB*	*FBN2*	*ITCH*	*NRAS*	*RPL36*	*TBXA2R*
ALDOA	*CLU*	*FCHO1*	*ITGA2*	*NSMCE2*	*RPL5*	*TBXAS1*
ALPI	*COG6*	*FCN1*	*ITGA2B*	*NT5C3A*	*RPL9*	*TCF3*
AMMECR1	*COL3A1*	*FCN2*	*ITGB2*	*NUP214*	*RPS10*	*TCIRG1*
AMN	*COL5A1*	*FCN3*	*ITGB3*	*OAS1*	*RPS14*	*TCN2*
ANK1	*COL5A2*	*FECH*	*ITK*	*OCA2*	*RPS15*	*TERC*
ANKRD26	*COLEC11*	*FERMT1*	*JAGN1*	*OFD1*	*RPS15A*	*TERF2*
ANO6	*COPA*	*FERMT3*	*JAK1*	*ORAI1*	*RPS17*	*TERF2IP*
AP1S3	*COX4I2*	*FGA*	*JAK2*	*OSTM1*	*RPS19*	*TERT*

AP3B1	*CPT2*	*FGB*	*JAK3*	*OTUD6B*	*RPS20*	*TF*
AP3D1	*CR2*	*FGG*	*KCNN4*	*OTULIN*	*RPS24*	*TFRC*
ARHGEF1	*CREBBP*	*FLI1*	*KDM1A*	*P2RY12*	*RPS26*	*TGFB1*
ARPC1B	*CRP*	*FLNA*	*KDM6A*	*PALB2*	*RPS27*	*TGFB2*
ATM	*CTC1*	*FOXE3*	*KDSR*	*PARN*	*RPS27A*	*TGFB3*
ATP6AP1	*CTLA4*	*FOXN1*	*KIF23*	*PAX5*	*RPS28*	*TGFBR1*
ATR	*CTNNBL1*	*FOXP3*	*KLF1*	*PC*	*RPS29*	*TGFBR2*
ATRX	*CTPS1*	*FPR1*	*KLKB1*	*PDHA1*	*RPS7*	*THBD*
B2M	*CTSC*	*FTCD*	*KMT2A*	*PDHX*	*RPSA*	*THPO*
BACH2	*CUBN*	*FYB1*	*KMT2D*	*PEPD*	*RRAS*	*THRA*
BCL10	*CXCR2*	*G6PC3*	*LAMTOR2*	*PFKM*	*RSPH1*	*THRB*
BCL11B	*CXCR4*	*G6PD*	*LARS2*	*PGM3*	*RSPH3*	*TICAM1*
BCO1	*CYB5A*	*GAS2L2*	*LAT*	*PIEZO1*	*RSPH4A*	*TINF2*
BLM	*CYB5R3*	*GAS8*	*LCK*	*PIH1D3*	*RSPH9*	*TIRAP*
BLNK	*CYBA*	*GATA1*	*LCT*	*PIK3R1*	*RTEL1*	*TLR3*
BLOC1S3	*CYBB*	*GATA2*	*LIG1*	*PKLR*	*RUNX1*	*TLR8*
BLOC1S5	*CYBC1*	*GBA*	*LIG4*	*PLA2G4A*	*SAMD9*	*TMC6*
BLOC1S6	*CYCS*	*GCLC*	*LIPA*	*PLAU*	*SAMD9L*	*TMC8*
BPGM	*CYP27A1*	*GFI1*	*LMAN1*	*PLCG2*	*SAMHD1*	*TMEM173*
BRCA1	*CSF2RB*	*GFI1B*	*LOX*	*PMM2*	*SAR1B*	*TMPRSS6*
BRCA2	*CSF3R*	*GGCX*	*LPIN2*	*PNP*	*SBDS*	*TNFAIP3*
BRIP1	*DBR1*	*GIMAP5*	*LRBA*	*POLA1*	*SBF2*	*TNFRSF11A*
BTK	*DCLRE1B*	*GINS1*	*LRRC6*	*POLD1*	*SEC23B*	*TNFRSF13B*
C15orf41	*DDX41*	*GLRX5*	*LRRC8A*	*POLD2*	*SEC61A1*	*TNFRSF13C*
C1GALT1C1	*DDX58*	*GNE*	*LZTR1*	*POLE*	*SEMA3E*	*TNFRSF4*
C1QA	*DEF6*	*GP1BA*	*LYST*	*POLE2*	*SERPINC1*	*TNFRSF9*
C1QB	*DGAT1*	*GP1BB*	*MAD2L2*	*POLR3A*	*SERPINE1*	*TNFSF11*
C1QBP	*DGKE*	*GP6*	*MAGT1*	*POLR3C*	*SERPINF2*	*TNFSF12*
C1QC	*DHFR*	*GP9*	*MALT1*	*POLR3F*	*SERPING1*	*TNXB*
C1R	*DIAPH1*	*GPI*	*MAN2B1*	*POMP*	*SF3B1*	*TOP2B*
C1S	*DKC1*	*GPR143*	*MANBA*	*POT1*	*SFTPB*	*TP53*
C3	*DNAAF1*	*GPX1*	*MAP2K1*	*PPP1CB*	*SFTPC*	*TPI1*
C3AR1	*DNAAF2*	*GSR*	*MAP2K2*	*PRF1*	*SH2B3*	*TPM4*

C4BPA	*DNAAF3*	*GSS*	*MAP3K14*	*PRG4*	*SH2D1A*	*TPP2*
C4BPB	*DNAAF4*	*GTF2H5*	*MAP3K8*	*PRKACG*	*SH3BP2*	*TRAC*
C5AR1	*DNAAF5*	*GUCY2C*	*MASP1*	*PRKCD*	*SH3KBP1*	*TRADD*
C5AR2	*DNAH1*	*HAVCR2*	*MASP2*	*PRKDC*	*SHOC2*	*TRAF3*
C6	*DNAH5*	*HAX1*	*MAST2*	*PRKG1*	*SI*	*TRAF3IP2*
C7	*DNAH9*	*HBA1*	*MASTL*	*PROC*	*SKI*	*TREX1*
C8A	*DNAI1*	*HBA2*	*MAT2A*	*PSEN1*	*SKIV2L*	*TRIM22*
C8B	*DNAI2*	*HBB*	*MBL2*	*PSENEN*	*SLC10A2*	*TRNT1*
C8G	*DNAJC21*	*HBD*	*MCFD2*	*PSMB8*	*SLC11A2*	*TSR2*
C9	*DNAL1*	*HBG1*	*MCIDAS*	*PSMG2*	*SLC19A1*	*TTC37*
CARD11	*DNASE1L3*	*HBG2*	*MCM4*	*PSTPIP1*	*SLC19A2*	*TTC7A*
CARD14	*DNASE2*	*HELLS*	*MECOM*	*PTGS1*	*SLC25A38*	*TUBA8*
CARD9	*DNMT3B*	*HFE*	*MED12*	*PTPN11*	*SLC26A3*	*TUBB1*
CARMIL2	*DOCK2*	*HK1*	*MEFV*	*PTPRC*	*SLC29A3*	*TYK2*
CASP10	*DOCK8*	*HMOX1*	*MFAP5*	*PTPRJ*	*SLC2A1*	*TYRP1*
CASP8	*DRC1*	*HOXA11*	*MLH1*	*PTX3*	*SLC2A10*	*UBE2T*
CAVIN1	*DTNBP1*	*HPS1*	*MLPH*	*PUS1*	*SLC30A10*	*UMPS*
CBL	*EFL1*	*HPS3*	*MOGS*	*RAB27A*	*SLC35A1*	*UNC119*
CBLIF	*EGLN1*	*HPS4*	*MPI*	*RAC2*	*SLC35C1*	*UNC13D*
CBS	*EIF2AK3*	*HPS5*	*MPIG6B*	*RACGAP1*	*SLC37A4*	*UNG*
CCBE1	*ELANE*	*HPS6*	*MPL*	*RAD50*	*SLC39A4*	*USB1*
CCDC103	*ENG*	*HRAS*	*MPO*	*RAD51*	*SLC39A7*	*VHL*
CCDC114	*EP300*	*HRG*	*MRAS*	*RAD51C*	*SLC45A2*	*VIPAS39*
CCDC39	*EPAS1*	*HSCB*	*MRE11*	*RAF1*	*SLC46A1*	*VKORC1*
CCDC40	*EPB41*	*HSPA9*	*MRTFA*	*RAG1*	*SLC4A1*	*VPS13B*
CCDC65	*EPB42*	*HYOU1*	*MS4A1*	*RAG2*	*SLC5A1*	*VPS33B*
CCNK	*EPCAM*	*ICOS*	*MSH2*	*RAP1A*	*SLC7A7*	*VPS45*
CCNO	*EPG5*	*ICOSLG*	*MSH6*	*RAP1B*	*SLC9A3*	*VPS4A*
CD19	*EPHB2*	*IFIH1*	*MTHFD1*	*RASA2*	*SLFN14*	*VSIG4*
CD247	*EPO*	*IFNAR1*	*MTR*	*RASGRP1*	*SLX4*	*VTN*
CD27	*EPOR*	*IFNAR2*	*MTRR*	*RASGRP2*	*SMAD3*	*VWF*
CD3D	*ERBIN*	*IFNGR1*	*MVK*	*RBCK1*	*SMAD4*	*WAS*
CD3E	*ERCC2*	*IFNGR2*	*MYD88*	*RBM8A*	*SMARCAL1*	*WDR1*

CD3G	*ERCC3*	*IKBKB*	*MYH11*	*RECQL4*	*SMARCD2*	*WIPF1*
CD40	*ERCC4*	*IKZF1*	*MYH9*	*REL*	*SNX10*	*WRAP53*
CD40LG	*ERCC6L2*	*IKZF5*	*MYLK*	*RELB*	*SP110*	*XK*
CD59	*ETV6*	*IL10*	*MYO5A*	*REN*	*SPAG1*	*XRCC2*
CD70	*EXTL3*	*IL10RA*	*MYSM1*	*RFWD3*	*SPINK5*	*YARS2*
CD79A	*F10*	*IL10RB*	*NAF1*	*RFX5*	*SPINT2*	*ZAP70*
CD79B	*F11*	*IL12B*	*NBAS*	*RFXANK*	*SPPL2A*	*ZBTB24*
CD81	*F12*	*IL12RB2*	*NBEAL2*	*RFXAP*	*SPRED1*	*ZCCHC8*
CD8A	*F13A1*	*IL17F*	*NBN*	*RHAG*	*SPTA1*	*ZMYND10*
